# Identification of DNA Repair-Related Genes Predicting Clinical Outcome for Thyroid Cancer

**DOI:** 10.1155/2022/8809469

**Published:** 2022-01-06

**Authors:** Ai-ying Zhang, Wei Li, Hai-yan Zhou, Jing Chen, Li-bin Zhang

**Affiliations:** ^1^Department of Clinical Laboratory, Affiliated Eye Hospital of Shandong University of Traditional Chinese Medicine, Eye Institute of Shandong University of Traditional Chinese Medicine, Jinan, Shandong, China; ^2^Department of General Surgery, Qingdao Eighth People's Hospital, Qingdao, Shandong, China; ^3^Department of Gynecology, Gaomi City Baicheng Center Hospital, Weifang, Shandong, China; ^4^Department of Endocrinology, Weifang Hanting District People's Hospital, Weifang, Shandong, China; ^5^Department of Endocrinology, The Second People's Hospital of Weifang, Weifang, Shandong, China

## Abstract

Recent studies have demonstrated the utility and superiority of DNA repair-related genes as novel biomarkers for cancer diagnosis, prognosis, and therapy. Here, we aimed to screen the potential survival-related DNA repair-related genes in thyroid cancer (TC). TCGA datasets were utilized to analyze the differentially expressed DNA repair-related genes between TC and nontumor tissues. The K–M approach and univariate analysis were employed to screen survival-related genes. RT-PCR was employed to examine the expression of DNA repair-related genes in TC samples and matched noncancer samples. CCK-8 analyses were used to determine cellular proliferation. Herein, our team discovered that the expression of four DNA repair-related genes was remarkably upregulated in TC samples in contrast to noncancer samples. Survival assays identified 14 DNA repair-related genes. In our cohort, we observed that the expression of TAF13 and DCTN4 was distinctly elevated in TC specimens in contrast to nontumor specimens. Moreover, knockdown of TAF13 and DCTN4 was observed to inhibit the TC cellular proliferation. Overall, the upregulation of TAF13 and DCTN4 is related to decreased overall survival in TC patients. Therefore, the assessment of TAF13 and DCTN4 expression may be useful for predicting prognosis in these patients.

## 1. Introduction

Thyroid cancer (TC) represents the most common endocrine malignancy, taking up 3.4% of the entire tumor diagnosis every year [1]. The transformation of thyroid follicle cells might cause the differentiation or undifferentiation of TC, via multiple steps which are the most adopted theories of follicle cell tumorigenesis [2]. Although some proofs have revealed that corpulency, smoking, hormone exposure, and some environmental pollution might be associated with TC, the only risk factor verified in TC is ionization radiation [3, 4]. The majority of TC sufferers at the early stage display beneficial prognoses posterior to thyroid resection and radioiodine. Nevertheless, the relapse is remarkably elevated when there is metastasis [5]. Therefore, finding new prognostic markers is critical for further treatment for TC.

Genome unsteadiness and the cumulation of variants are signatures of tumor development [6]. The anticipated cell reaction to DNA damages which cannot be restored is cellular death through aging or programmed cell death [7]. Various proteins at present are known to exert a pivotal impact on sustaining DNA integrity, especially with the identification and repairment of DNA damages via several signal paths which appear greatly conserved in terms of biology [8, 9]. In recent years, more and more DNA repair gene alterations have exhibited a vital modulatory function in the developmental process of various tumors [10, 11]. In recent years, researchers have determined genome flaws in DNA repairment in the late period and primary TC, which has given rise to researchers clinically providing a potent reason to develop PARP suppressors and DNA-damage agents within such molecule-level TC subtype [12–14]. In addition, several DNA repair and replication-related gene signatures that could predict the prognosis and progression of tumors have been developed [15, 16]. However, the expression and function of DNA repair-related genes in TC were rarely reported.

In this study, we analyzed TCGA datasets and identified four dysregulated DNA repair-related genes in TC. In addition, we also identified 14 survival-related DNA repair-related genes in TC. Then, we chose six genes for further confirmation using 10 pairs of TC specimens and nontumor specimens from our cohort. Our findings focused on the possibility of DCTN4 and TAF13 utilized as new markers for TC.

## 2. Materials and Methods

### 2.1. Patients and Clinical Samples

TC samples and neighboring noncancer samples from sufferers who had undergone curative resection were collected between July 2020 and June 2021 from The Second People's Hospital of Weifang. All tissues were histopathologically confirmed by two experienced pathologists. No sufferers underwent chemotherapy, radiotherapy, or immunotherapy prior to surgeries. Cancer samples and neighboring healthy samples were harvested and reserved under −80°C for later assays. Written informed consent for the analysis of tissue specimens was obtained from all patients.

### 2.2. Data Collection

Genetic expression quantitation data and relevant clinic features of TC sufferers were acquired from the TCGA datasets (http://portal.gdc.cancer.gov/). The DNA damages and DNA repairment-associated genetic lists were acquired from GSEA genetic sets via the key word “DNA AND damage” or “DNA AND repair.” Eventually, 150 genes associated with DNA damages and repairment were involved in the analyses. By comparing thyroid carcinoma tissues to normal tissues and using R package edgeR in R software (version 3.4.1), differentially expressed genes were identified with thresholds |log2FoldChange| > 2 as well as adjusted *P* < 0.05.

### 2.3. Cox Regression and Survival Analyses

The TCGA specimens (*n* = 510) were separated into a high-expression group and low-expression group via the medium expressing level of every single candidate dysregulated DNA repair-related genes as the threshold. Univariate prognostic analyses and K–M analyses were afterwards finished for these two groups via the “survival” package of R program. To illustrate the intersection between dysregulated DNA repair-related genes and prognostic DNA repair-related genes, a Venn diagram program was employed.

### 2.4. Cell Lines and Transfection

Four mankind TC lineage cells (TPC, BHP5-16, K1, and BHP2-7) and mankind thyroid follicle epithelia (Nthy-ori 3-1) were acquired from the Type Culture Collection of the Chinese Academy of Sciences. The entire cells were maintained in DMEM (Gibco, America) in moist atmosphere with 5% carbon dioxide under 37°C. Such intermediary involved 10% FBS (Hyclone, America) and 1% penicillin/streptomycin.

DCTN4 and TAF13 expressions were knocked down by transiently transfecting TC cells with DCTN4‐specific siRNA (si-DCTN4) or TAF13-specific siRNA (si-TAF13). In short, siRNAs were introduced into the cells via transfection by virtue of liposome transfection 2000 for 48 h; they were afterwards cultivated for later assays.

### 2.5. Quantitative Reverse-Transcription PCR (qRT-PCR)

The overall RNA from TC samples and cells was abstracted via TRIzol® reagent (Invitrogen, America), and 200 ng abstracted RNA was converted to cDNA via reverse transcription through the ReverTra Ace qPCR RT Kit (Toyobo, Japan) prior to qRT-PCR. The qRT-PCR was employed to identify comparative RNA level, which was determined via a 7900 RealTime PCR System through the SDS 2.3 program sequence identification system (Applied Biosystem, America) by virtue of the SYBR Green (Takara) approach. The comparative expressing levels of mRNAs were evaluated via the 2^−ΔΔCq^ approach, with GAPDH as the internal reference. The primers are presented in Table 1.

### 2.6. Cell Proliferation Assay

TPC and BHP2-7 cells were inoculated into 96-well dishes (1 × 10^3^ cells/well) and cultivated with 100 *μ*l intermediary involving 10% FBS. Posterior to cellular transfection, they were cultivated for 0, 24, 48, and 72 h, before cultivation in 10 *μ*l CCK-8 liquor (CK04, Dojindo, Yanhui Technology, Jiading, Shanghai, China) under 37°C or 60 min. The optical density was identified at 450 nm via a microplate reading device.

### 2.7. Statistical Analysis

The entire calculation was finished via the SPSS 17.0 (IBM, America) or R software, version 3.6.3. The diversity between these groups was studied via Student's *t*-test. The K–M approach was employed to draw the survival curves for prognosis analysis, and the log-rank test was leveraged to speculate the significance on statistics. The Cox proportion risk model was employed to identify the prognostic value of genes in TC. A *P* < 0.05 was deemed to be statistically significant.

## 3. Results

### 3.1. Determination of the Dysregulated DNA Repair-Associated Genes in TC

**Figure 1:**
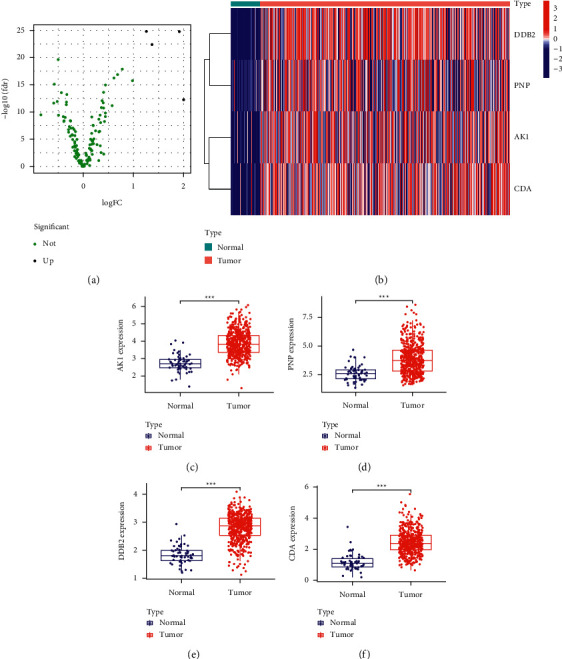
The dysregulated DNA repair-related genes in TC. (a) Aberrant expression DNA repair-related genes in TC tissues were reflected by the volcano plot. (b) Layer clustering analyses of differential expression DNA repair-related genes (fold change > 2; *P* < 0.05) in TC and healthy samples. (c–f) The expression of AK1, PNP, DDB2, and CDA was remarkably elevated in TC samples in contrast to healthy specimens. ^*∗∗∗*^*P* < 0.001.

To identify the dysregulated DNA repair-associated genes in TC, we downloaded the list of DNA repair-associated genes from GSEA, and 135 genes were screened. Then, we analyzed TCGA datasets and identified 4 dysregulated DNA repair-related genes in TC including AK1, PNP, DDB2, and CD1 (Figure 1(a)). The expressing pattern of the abovementioned four genes was shown in heatmap (Figure 1(b)). In addition, we found the expression of AK1 (Figure 1(c)), PNP (Figure 1(d)), DDB2 (Figure 1(e)), and CDA (Figure 1(f)) was remarkably elevated in TC samples in contrast to healthy specimens. Our findings suggested them as functional regulators in TC progression.

### 3.2. Determination of the DNA Repair-Associated Genes with Potential Prognostic Value in TC

**Figure 2:**
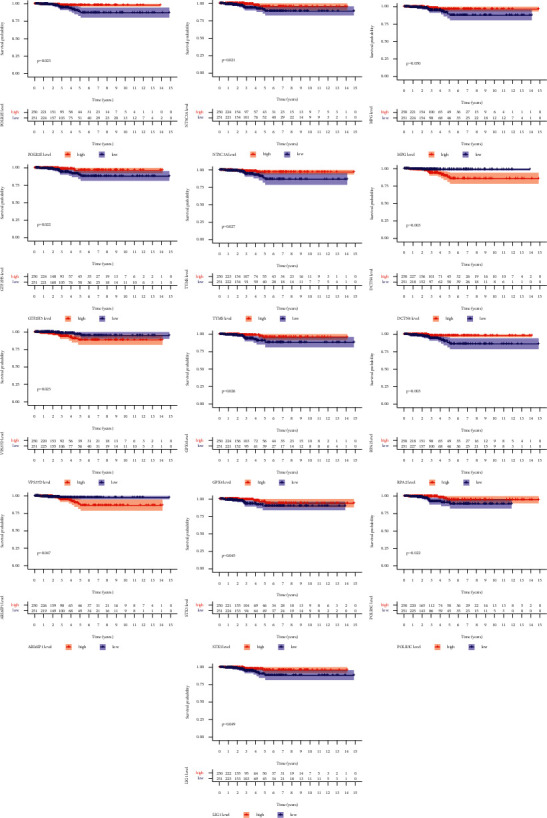
The K–M approach was employed to screen the survival-related DNA repair-related genes in TC.

**Figure 3:**
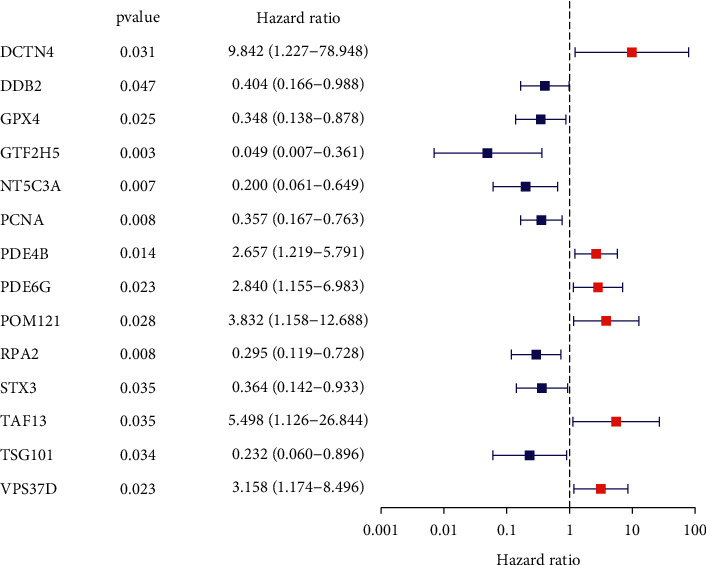
Univariate analysis of the 135 repair-related genes in TC.

To screen prognostic DNA repair-related genes, we performed the Kaplan–Meier method based on TCGA datasets and identified 13 genes, including ARL6IP1, DCTN4, GPX4, GTF2H5, LIG1, MPG, NT5C3A, POLR2E, POLR3C, RPA2, STX3, TYMS, and VPS37D (Figure 2). In addition, we also performed univariate analysis which revealed that high expression of DCTN4, PDE4B, PDE6G, POM121, TAF13, and VPS37D and low expression of DDB2, GPX4, GTF2H5, NT5C3A, PCNA, RPA2, STX3, and TSG101 were associated with survivals of TC patients (Figure 3). These findings provided a new clue for the identification of novel prognostic biomarkers in the section of DNA repair-associated genes.

### 3.3. The Distinct Upregulation of TAF13 and NCTN4 in TC and Their Oncogenic Roles

**Figure 4:**
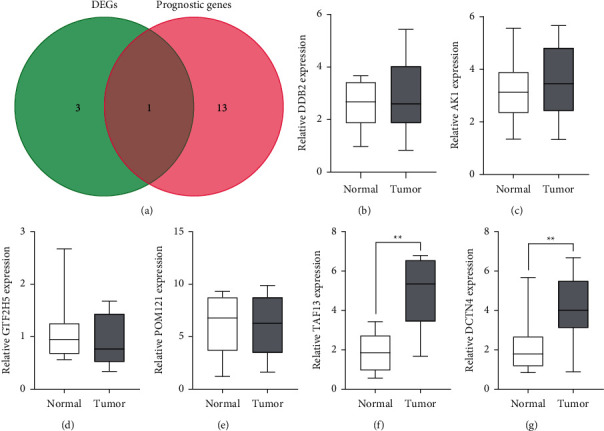
The distinct upregulation of TAF13 and DCTN4 in TC. (a) Venn diagram software of the common genes with dysregulated expression and potentially prognostic value in TC. (b–g) RT-PCR for the expressions of (b) DDB2, (c) AK1, (d) GTF2H5, (e) POM121, (f) TAF13, and (g) DCTN4. ^*∗∗*^*P* < 0.01.

**Figure 5:**
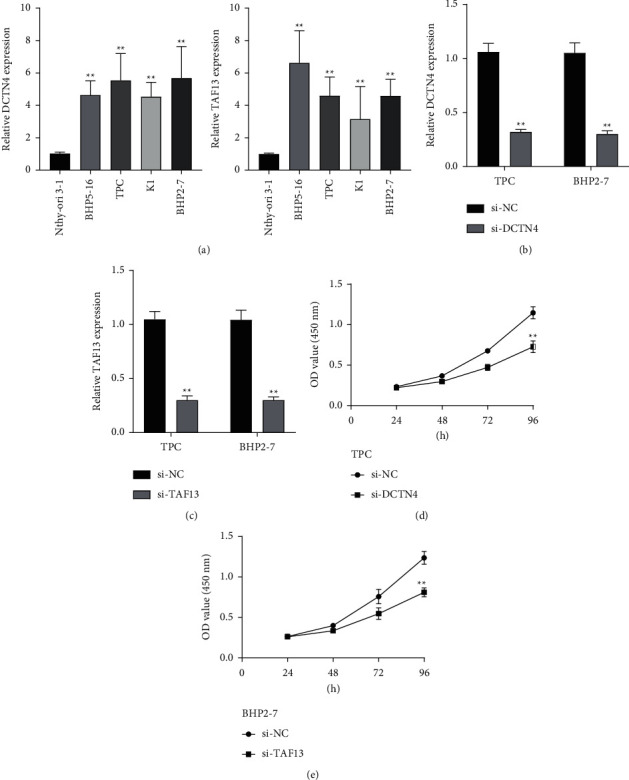
The oncogenic roles of DCTN4 and TAF13 in TC. (a) qRT-PCR analyses of DCTN4 and TAF13 expressing levels in four TC lineage cells (TPC, BHP5-16, K1, and BHP2-7) compared with the mankind thyroid follicular epithelial cells (Nthy-ori 3-1). (b, c) qRT-PCR analyses of DCTN4 and TAF13 expressions following treatment of TPC and BHP2-7 cells with siRNA targeting DCTN4 or TAF13. (d) CCK-8 analysis was performed to identify cellular proliferation. ^*∗∗*^*P* < 0.01.

Then, we used Venn Diagram which confirmed DDB2 as a dysregulated DNA repair-related gene which had potentially prognostic value in TC (Figure 4(a)). Then, we performed RT-PCR to explore its expression, finding that DDB2 was not differentially expressed between TC specimens and nontumor specimens (Figure 4(b)). In addition, we chose AK1, GTF2H5, POM121, TAF13, and DCTN4 for further study. As shown in Figures 4(c)–4(e), the expression of AK1, GTF2H5, and POM121 between TC specimens and nontumor specimens remained unchanged. However, we discovered that the expressions of TAF13 and DCTN4 were distinctly elevated in TC specimens in contrast to matched nontumor specimens (Figures 4(f) and 4(g)). Moreover, high expression of TAF13 and DCTN4 was also observed in BHP5-16, TPC, K1, and BHP2-7 in contrast to nthy-ori 3-1 (Figure 5(a)). To investigate the potential role of TAF13 and DCTN4 in TC cells, our team used siRNA to decrease their levels in TPC and BHP2-7, which was confirmed by RT-PCR (Figures 5(b) and 5(c)). Finally, CCK-8 assays revealed that knockdown of TAF13 and DCTN4 distinctly suppressed the proliferation of TC cells (Figures 5(d) and 5(e)).

## 4. Discussion

There have been some developments in the therapies of TC over the past few decades [17]. Such development is facilitated by the progression in diagnosis and treatment modalities and new molecule-level target treatment [18]. Further endeavors are required to realize satisfactory prognostic results in this regard, which remains daunting. Clinical management highlights the significance of timely and valid identification and forecast of prognostic results, so as to achieve personalized therapies [19, 20]. The usage of prognosis models is helpful to guide decision making clinically and is pivotal for precise medical treatment [21, 22]. Given the important roles of DNA repair-related genes in tumor development, it is necessary to screen survival-related DNA repair-related genes.

Recently, epidemiology researchers have revealed that 2/3 tumors are induced by DNA replicational errors [23]. Particularly, errors in mRNA replications, such as the variant in the inhibitor gene P53, are especially vital for the tumor progression [24, 25]. In this study, we identified four dysregulated DNA repair-related genes, including AK1, PNP, DDB2, and CDA. Previously, several studies have reported the tumor-related function of the abovementioned four genes in different cancer types; e.g., DDB2 was reported to be greatly expressed in ovarian cancer and suppressed ovarian tumor cell dedifferentiation by suppressing ALDH1A1 [26]. CDA polymorphisms are found to be associated with clinical outcomes in gastroenteric cancer patients treated with capecitabine-based chemotherapy [27]. Then, we identified 14 prognostic DNA repair-related genes. However, we just found one gene DDB2 which exhibited a high level in TC and predicted a poor prognosis. DDB2 may be a novel biomarker for TC.

Then, we chose six genes for further confirmation, including DDB2, AK1, GTF2H5, POM121, TAF13, and DCTN4. RT-PCR assay revealed that DDB2 expression remained unchanged between TC specimens and nontumor specimens, which was not consistent with the abovementioned results. Importantly, we observed that TAF13 and DCTN4 expression was distinctly elevated in TC samples in contrast to paired noncancer samples. TAF13 produces a histone-fold-like heterodimer with TAF11, and such heterodimer is pivotal for the recruiting into the RNA polymerase II general TFIID protein complex [28]. To date, the expression and function of TAF13 were rarely reported. We observed that knockdown of TAF13 remarkably inhibited the TC cellular proliferation. Previous studies discovered that the DCTN family was related to several neurodegeneration illnesses [29]. DCTN4 belonged to the DCTN family. Previously, DCTN4 was reported to be associated with poor prognosis of colon adenocarcinoma and low-grade glioma [30, 31]. In addition, our team discovered the knockdown of DCTN4 in the TC cellular proliferation. Our findings provided a new clue for the determination of prognostic biomarkers for TC.

## 5. Conclusions

We identified 14 prognostic DNA repair-related genes and provided evidence that DCTN4 and TAF13 may serve as a tumor promotor in TC. The results herein elucidated an underlying causal link beneath the oncogenesis effect of DCTN4 and TAF13 in TC and revealed that DCTN4 and TAF13 could be a prospective biomarker and underlying treatment target for TC.

## Figures and Tables

**Table 1:** The primers used in this study for RT-PCR.

Names	Sequences (5′−3′)
AK1: F	GAAGAGTTTGAGCGACGGATT
AK1: R	CAGCCGCTTTTTGATGGTCTC
GTF2H5: F	AAGACATTGATGACACTCACGTC
GTF2H5: R	GGGAAAAAGCATTTTGGTCCATT
POM121: F	GCCTTTGTCCAGTCGGTTTG
POM121: R	TTGATGAGCGGAATAGCTTGC
TAF13: F	AGAAGACCCCACGTTTGAGGA
TAF13: R	TTGCCTTGTGAGTCATTTCAGT
DCTN4: F	CACACCCTCTCTACTCGGG
DCTN4: R	ACATGCCAGGTAATAGGCTTTC
DDB2: F	ACCTCCGAGATTGTATTACGCC
DDB2: R	TCACATCTTCTGCTAGGACCG
GAPDH: F	GGAGCGAGATCCCTCCAAAAT
GAPDH: R	GGCTGTTGTCATACTTCTCATGG

## Data Availability

The analyzed datasets generated during the study are available from the corresponding authors on reasonable request.
